# Seewis virus, a genetically distinct hantavirus in the Eurasian common shrew (*Sorex araneus*)

**DOI:** 10.1186/1743-422X-4-114

**Published:** 2007-10-30

**Authors:** Jin-Won Song, Se Hun Gu, Shannon N Bennett, Satoru Arai, Maria Puorger, Monika Hilbe, Richard Yanagihara

**Affiliations:** 1 Department of Microbiology, College of Medicine, and Institute for Viral Diseases, Korea University, 5-ga, Anam-dong, Sungbuk-gu, Seoul 136-705, Korea; 2 Departments of Tropical Medicine and Medical Microbiology and of Pediatrics, John A. Burns School of Medicine, University of Hawai'i at Manoa, 651 Ilalo Street, Honolulu, HI 96813, USA; 3 Institute of Veterinary Pathology, University of Zurich, Winterthurerstr. 268, 8057 Zürich, Switzerland

## Abstract

More than 20 years ago, hantaviral antigens were reported in tissues of the Eurasian common shrew (*Sorex araneus*), Eurasian water shrew (*Neomys fodiens*) and common mole (*Talpa europea*), suggesting that insectivores, or soricomorphs, might serve as reservoirs of unique hantaviruses. Using RT-PCR, sequences of a genetically distinct hantavirus, designated Seewis virus (SWSV), were amplified from lung tissue of a Eurasian common shrew, captured in October 2006 in Graubünden, Switzerland. Pair-wise analysis of the full-length S and partial M and L segments of SWSV indicated approximately 55%–72% similarity with hantaviruses harbored by Murinae, Arvicolinae, Neotominae and Sigmodontinae rodents. Phylogenetically, SWSV grouped with other recently identified shrew-borne hantaviruses. Intensified efforts are underway to clarify the genetic diversity of SWSV throughout the geographic range of the Eurasian common shrew, as well as to determine its relevance to human health.

## Findings

Viruses antigenically related to Hantaan virus (HTNV), the prototype virus of hemorrhagic fever with renal syndrome, have been isolated from the Asian house shrew (*Suncus murinus*), greater white-toothed shrew (*Crocidura russula*) and Chinese mole shrew (*Anourosorex squamipes*) [1-4], indicating that shrews are capable of serving as incidental hosts of hantaviruses typically harbored by rodents. Insectivores, or soricomorphs, also appear to harbor genetically distinct hantaviruses, as evidenced by the recent demonstration of Camp Ripley virus (RPLV) in the northern short-tailed shrew (*Blarina brevicauda*) [5], Cao Bang virus (CBNV) in the Chinese mole shrew [6], Tanganya virus (TGNV) in the Therese shrew (*Crocidura theresae*) [7], and Ash River virus and Jemez Springs virus in the masked shrew (*Sorex cinereus*) and the dusky shrew (*Sorex monticolus*), respectively [8]. Moreover, Thottapalayam virus (TPMV), a previously unclassified virus isolated from the Asian house shrew [9,10], is now known to be a bona fide shrew-borne hantavirus [11-14].

Earlier reports of hantaviral antigens in tissues of the Eurasian common shrew (*Sorex araneus*), alpine shrew (*Sorex alpinus*), Eurasian water shrew (*Neomys fodiens*) and common mole (*Talpa europea*), captured in European Russia and the former Yugoslavia [15-17], have largely gone unnoticed. In this short report, we present the genetic and phylogenetic analyses of a new hantavirus, designated Seewis virus (SWSV), detected in the Eurasian common shrew. These findings add to the expanding database on soricid-associated hantaviruses and forecast that many more hantaviruses will be found in diverse shrew species throughout Eurasia.

Liver tissue from three *Crocidura leucodon *and lung tissue from one *Neomys anomalus *and five *Sorex araneus*, captured during August and October 2006, in the village of Seewis (46°59'N, 9°38'E), located in the Swiss canton of Graubünden, a region endemic for Borna disease located approximately 130 kilometers east of Zurich [18], were studied. Total RNA, extracted from 20–50 mg of each tissue, using the RNA-Bee™ isolation kit (TEL-TEST, Inc., Friendswood, TX), was reverse transcribed, using M-MLV reverse transcriptase (Promega, Madison, WI) and a conserved primer (OSM55: 5'-TAGTAGTAGACTCC-3' and SWS S1093F: 5'-TACAGCTGAGGAGAAGC-3' for the S segments; OSM55 and SWS L1351F: 5'-CAAGGCCCAGCAAAACATAC-3' for the L segment; and OSV697: 5'-GGACCAGGTGCADCTTGTGAAGC-3' for the M segment).

Gene-amplification reactions were performed in 50-μL mixtures, containing 200 μM dNTP, 0.5 U of super-therm polymerase (PureTech Co., Ltd. Seoul, Korea), 1 μg of cDNA and 10 pM of each primer. Oligonucleotide primer sequences for nested PCR, designed from TPMV and other hantaviruses, were OSM55 and OSV845: 5'-CTTAGCTCGGGATCCATRTC-3', then OSM55 and OSV847: 5'-TATCATCACCMAGRTGGAA-3', SWS S1130F: 5'-TACCAATCTTATCTGCGTC-3' and CBS-3'endR: 5'-TAGTAGTAKRCTCCYTRAA-3' for the S segment; OSV697 and T-M1485R: 5'-CCAGCCAAARCARAATGT-3', then T-M1199F: 5'-TAAVTTCAMCAAC ATGTCT-3' and T-M1485R for the M segment; and OSM55 and T-L1454R: 5'-ATGCCC WATATGCCATGC-3', then OSM55 and T-L390R: 5'-GTCACWGTRACCTC-3', MJN L181F: 5'-ATGAGATGATAAARCATGA-3' and T-L1454R, SWS L1351F and PHL 3445R: 5'-GRTTAAACATACTCTTCCTCCACATCTC-3', then SWS L1351F and SWS L2180R: 5'-GTA ACCTCAGATATCAAGC-3' for the L segment. Initial denaturation was at 94°C for 5 min, followed by touchdown cycling with denaturation at 94°C for 40 sec, annealing from 50°C to 37°C for 40 sec, elongation at 68°C for 1 min 20 sec, then 25 cycles of denaturation at 94°C for 40 sec, annealing at 40°C for 40 sec and elongation at 68°C for 1 min 20 sec in a Mastercycler ep gradient S (Eppendorf AG, Hamburg, Germany). PCR products were purified by the Wizard PCR Preps DNA Purification System (Promega), and DNA sequences of at least three clones of each amplicon were determined in both directions, using the dye primer cycle sequencing ready reaction kit (Applied Biosystems, Foster City, CA) on an automated sequencer (Model 377, Perkin Elmer Co.) [19]. DNA sequences were then aligned using Clustal W [20] and transAlign [21] with publicly available hantavirus sequences and analyzed phylogenetically by PAUP version 4.0 [22] and RAxML [23]. The maximum likelihood (ML) method under the GTR+I+G model of evolution, as selected by Modeltest v.3.7 [24], was used, and ML bootstrap support was generated using the RAxML web-server prototype that implements a novel rapid bootstrapping algorithm [25].

Host identification was confirmed by mitochondrial DNA (mtDNA) sequencing. Briefly, the cytochrome *b *region of mtDNA was amplified by PCR, using previously described universal primers, which permit amplification of a 482-bp product (L14115: 5'-CGAAGCTTGATATGA AAAACCATCGTTG-3'; L14532: 5'-GCAGCCCCTCAGAATGATATTTGTCCAC-3') [26].

Unique hantavirus sequences of the S, M and L segments were amplified from lung tissue of a single *S. araneus*. The full-length S-genomic segment of SWSV strain mp70 was 1,641 nucleotides, with a predicted nucleocapsid protein of 429 amino acids, starting at nucleotide position 40, and a 314-nucleotide 3'-noncoding region. Pair-wise alignment and comparison of the coding region of the S segment indicated genetic similarities of 55.3–58.1% and 55.8–61.0% at the nucleotide and amino acid levels, respectively, from representative Murinae, Arvicolinae, Neotominae and Sigmodontinae rodent-borne hantaviruses. Unexpectedly, SWSV was even less similar to TPMV VRC-66412 (49.3% at the nucleotide and 44.2% at the amino acid level). As in other soricid-borne hantaviruses discovered to date, the hypothetical NSs opening reading frame, typically found in Arvicolinae and Neotominae rodent-borne hantaviruses, was not found in SWSV.

Analysis of a 250-nucleotide region of the Gn glycoprotein-encoding M segment also exhibited low nucleotide sequence similarity to rodent-associated hantaviruses, including HTNV 76–118 (68.8%), Dobrava virus (DOBV) AP99 (69.9%), Soochong virus (SOOV) SC-1 (72.4%), Seoul virus (SEOV) HR80-39 (72.0%), Puumala virus (PUUV) Sotkamo (65.0%), Tula virus (TULV) M5302v (66.4%) and Sin Nombre virus (SNV) NMH10 (68.8%). And comparison of a 3,327-nucleotide region of the L segment showed similar degrees of sequence identity of approximately 65% between SWSV and rodent-borne hantaviruses.

**Figure 1:**
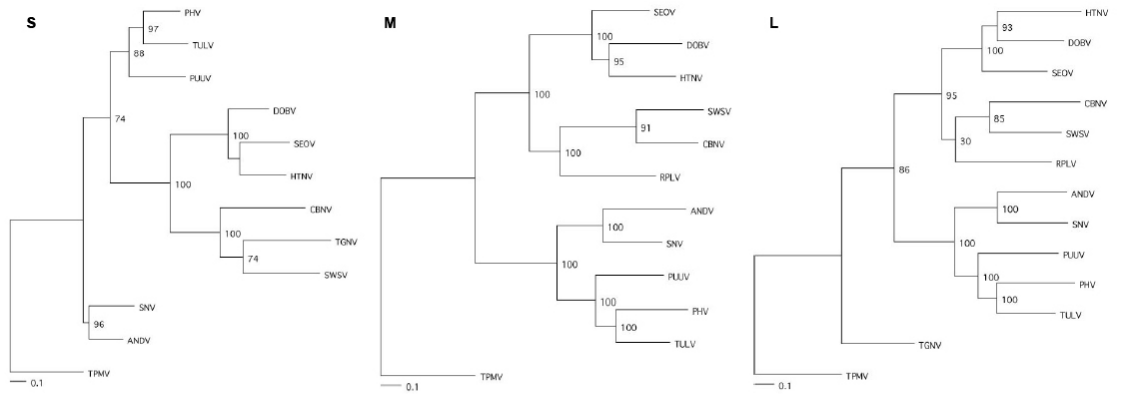
**Phylogenetic relationships between Seewis virus (SWSV) and representative rodent- and soricid-borne hantaviruses, using the GTR+I+G model of evolution**. Maximum likelihood phylogenies, based on full-length coding alignments of S, M and L segments, incorporating 1,290, 250 and 3,300 nucleotides, respectively, of SWSV strain mp70, shown in relationship to representative Murinae rodent-borne hantaviruses, including Hantaan virus (HTNV 76–118, NC_005218, NC_005219, NC_005222), Dobrava virus (DOBV Greece, NC_005233, NC_005234, NC_005235), and Seoul virus (SEOV 80–39, NC_005236, NC_005237, NC_005238); Arvicolinae rodent-borne hantaviruses, including Tula virus (TULV M5302v, NC_005227, NC_005228, NC_005226), Prospect Hill virus (PHV PH-1, Z49098, X55128, EF646763) and Puumala virus (PUUV Sotkamo, NC_005224, NC_005223, NC_005225); and Sigmodontinae and Neotominae rodent-borne hantaviruses, including Andes virus (ANDV Chile 9717869, NC_003466, NC_003467, NC_003468) and Sin Nombre virus (SNV NMH10, NC_00521, NC_005215, NC_005217). Also included are Camp Ripley (RPLV MSB89863, EF540771, EF540774) from the northern short-tailed shrew (*Blarina brevicauda*), Cao Bang virus (CBNV TC-3, EF543524, EF543525, EF543526) from the Chinese mole shrew (*Anourosorex squamipes*), Tanganya virus (TGNV Tan826, EF050454, EF050455) from the Therese shrew (*Crocidura theresae*), and Thottapalayam virus (TPMV VRC-66412, AY526097, EU001329, EU001330) from the Asian house shrew (*Suncus murinus*). The numbers at each node are bootstrap support values (expressed as the percentage of replicates in which the node was recovered), as determined for 100 ML replicates using RAxML [25]. The scale bar indicates 0.1 nucleotide substitutions per site. GenBank accession numbers for SWSV: S (EF636024); M (EF636025) and L (EF636026).

Phylogenetic analyses, based on the full-length S and partial M and L segments, generated by the ML method, indicated that SWSV was distinct from rodent-borne hantaviruses (Figure 1). In the representative ML trees, based on M- and L-segment sequences, SWSV clustered with CBNV and RPLV, two recently identified hantaviruses harbored by soricine shrews. S-segment analysis further supported the close relationship between SWS and CBNV. While TPMV was consistently more divergent phylogenetically from SWSV than even the rodent-borne hantaviruses, TGNV was not (Figure 1). In such trees, SWSV, although distinct, shared a more recent common ancestor with Murinae rodent-borne hantaviruses than with TPMV, suggesting a possible host-switching event in the distant past.

RT-PCR amplification of SWSV presented unanticipated challenges. While still cool on arrival, the small amount of tissue from the single infected shrew had thawed in transit, resulting in a low RNA yield, which limited the options in cDNA synthesis. Also, the divergent genome of SWSV made difficult the designing of suitable primers. Although these difficulties were partly overcome, they contributed to our inability to obtain the full genome of SWSV. Future work will be necessary to complete this task and to isolate SWSV in cell culture.

Although several viruses have been isolated from soricomorphs, including Erve virus from *Crocidura russula *[27], tick-borne encephalitis virus from *Talpa europea *[28] and *S. araneus *[29], and a canine distemper-like paramyxovirus from *Erinaceus europeus *[30], their detection has been largely incidental or accidental. By contrast, our study specifically targeted the identification of new hantaviruses in shrews, as a means of better understanding their evolutionary origins. In some ways, however, the demonstration of a phylogenetically distinct hantavirus in the Eurasian common shrew was not surprising and was thoroughly consistent with decades-old reports of hantavirus antigens in *S. araneus *in the former Yugoslavia and Russia [15-17]. The important distinction is that we now have sequence data to substantiate the existence of a hantavirus in the Eurasian common shrew. Further support for this long co-evolutionary relationship is provided by the recent detection of SWSV sequences in *S. araneus *captured in Hungary and Finland (S. Arai and R. Yanagihara, unpublished observations).

Found in woodlands, grasslands and hedgelands throughout Northern Europe, including Scandinavia and Great Britain (but excluding Ireland), and extending throughout Russia, *S. araneus *(family Soricidae, subfamily Soricinae) is among the most widely dispersed small mammal species in Eurasia. Although their nests are generally made underground or under dense vegetation, they occasionally occupy burrows of mice, voles and moles. While solitary, their extremely aggressive territorial behavior and carnivorous eating habits make plausible the acquisition and transmission of hantavirus infection through wounding. However, to what extent such host-switching events might have occurred in the distant past is unknown. Nevertheless, as judged by the nucleotide sequence analyses of the S-, M- and L-genomic segments, the polyphylogenetic relationships of SWSV and other soricid- and rodent-associated hantaviruses are suggestive. The discovery and genetic characterization of other soricid-borne hantaviruses will clarify whether the reservoir host of the primordial hantavirus was harbored by a soricid or rodent ancestor.

As recently estimated from sequence analysis of cytochrome *b *mtDNA and nuclear genes BRCA1 and ApoB, the Palearctic and Nearctic Soricinae (i.e., Sorex in Eurasia and Otisorex in North America) diverged approximately 14 million years before present [31]. In this regard, the identification of genetically distinct hantaviruses in North American soricines, such as *S. cinereus *and *S. monticolus *in the United States [8], will aid in the elucidation of the phylogeography and parallel co-evolution of *Sorex*-borne hantaviruses in the Old and New Worlds. Also, intensified efforts to isolate SWSV and other hantaviruses harbored by shrews will clarify their importance to human health.

## Competing interests

The author(s) declare that they have no competing interests.

## Authors' contributions

JWS coordinated the implementation of the project, including the design of oligonucleotide primers and optimization of gene-amplification reactions. SHG performed the RNA extraction, RT-PCR and DNA sequencing. SNB performed the sequence and phylogenetic analyses. SA participated in the design of RT-PCR primers. MP and MH provided tissues and background data of wild-caught shrews. RY conceived the study design, arranged the collaboration and provided oversight. All authors contributed to the preparation of the manuscript.
